# Allene chemistry

**DOI:** 10.3762/bjoc.7.50

**Published:** 2011-04-07

**Authors:** Kay M Brummond

**Affiliations:** 1 University of Pittsburgh, Department of Chemistry, Chevron Science Center, 219 Parkman Avenue Pittsburgh, PA 15260, USA

## Allenes: The changing landscape of C_sp_

As organic chemists, we continually search for transformations to produce compounds in increasingly efficient and expedient ways, and to synthesize new compounds possessing interesting properties. One particularly appealing strategy is to exploit underutilized functional groups. By using this strategy, novel arrays of atoms that were previously unattainable are made possible. There are hurdles to overcome with this approach, that include, but are not limited to, an incomplete understanding of functional group compatibility issues that may arise during their preparation and/or that may be caused by their presence in subsequent reactions. The focus of this Thematic Series of the *Beilstein Journal of Organic Chemistry* is allenes, which even after decades of use can still be classified as an underutilized functional group. However, great advances in the chemistry of allenes have been made since the time when they were thought to be, “difficult to prepare and very reactive, and not commonly encountered ...” [1].

I first became interested in allenes as a graduate student with Professor Ray Funk at Pennsylvania State University while working on the preparation of ene–diyne systems. Interestingly, I always seemed to isolate allenes instead of the compounds I was hoping to obtain. My interests in allenes continued in my first faculty position at West Virginia University, where my colleagues Professors Bob Moore (of the Doering*-*Moore*-*Skattebøl reaction) and Kung Wang (contributor to this issue) contributed greatly to my appreciation and understanding of the allene functional group.

Focusing on a particular functional group at first glance may seem trivial and obvious, but the importance of these types of studies is underscored by the fact that numerous Nobel prizes have been awarded for the study “alkenes in synthesis”. More importantly, transformations involving allenes can provide new scaffolds that lead to an expansion of chemical space where currently nearly half of all existing organic compounds can be described by a mere 143 molecular frameworks [2]. This data supports the notion that we tend towards compounds and functionalities with which we are familiar and understand, but this, nevertheless, creates a limitation to our knowledge of our field.

This Thematic Series entitled “Allene chemistry” in the *Beilstein Journal of Organic Chemistry* represents contributions from leading chemists exploring a wide range of reactions involving allenes as reactants or products. The articles within detail discoveries surrounding the allene group and provide knowledge that chemists need in order to make informed decisions about whether or not to include an allene in a synthetic plan. I would like to thank all the authors for their contributions to this issue.

Kay Brummond

Pittsburgh, April 2011
